# A Case of Tetanus Following Compound Fracture of the Leg: Recovery

**Published:** 1870-04

**Authors:** E. J. A. Trimble, David McDill

**Affiliations:** Sagetown, Ill.; Biggsville, Ill.


### CHICAGO MEDICAL EXAMINER.

N. S. DAVIS, M.D., Editor.

VOL. XI. APRIL, 1870. NO. 4.

### Original Contributions.

ARTICLE XI.

### A CASE OF TETANUS FOLLOWING COMPOUND FRACTURE OF THE LEG: RECOVERY.

Under the care of E. J. A. TRIMBLE, M.D., of Sagetown, Ill., and DAVID McDILL, M.D., of Biggsville, Ill.

REPORTED BY DR. TRIMBLE.

On the 8th day of December last, Edward Gotke, a laborer in the stone quarry at this place, while engaged, with several others, in moving a heavy piece of rock, had his right leg caught between the same and a bank of frozen earth, and severely injured. On examination, about two hours after the accident occurred, a fracture of both the tibia and fibula was discovered in the lower portion of the middle third of the leg, both bones being broken on the same line, and in a transverse direction, but with no comminution of the fragments, and no displacement. About two inches above the point of fracture, on the posterior surface of the leg, and a little to the inner side, was a wound of an inch in length, externally, communicating with the fragments; while the skin and underlying tissues surrounding the injured bones, were badly crushed and bruised from a point two inches below the seat of injury, to one four inches above. Extensive discoloration existed between these two points, while the cellular tissue throughout almost the entire length of the limb, from knee to ankle, was infiltrated with air in such a degree as to present marked resonance on percussion; there had been considerable hemorrhage, but the principal vessels of the leg were apparently uninjured.

Dr. McDill agreed with me in thinking that the probability of our being able to save the limb was not great, but the solicitations of the patient and his friends finally induced us to make an attempt to do so. The leg was accordingly laid in a fracture box, supported by bags of cotton, and cold lotions constantly applied, a little morphia being given occasionally to prevent muscular contraction. By the third day, the swelling of the limb had mostly subsided, and sloughing of the integument begun. The pads of cotton were now removed and replaced with bran, which could be removed from about the point of injury whenever it had become saturated with the discharge; and the sloughing surface frequently washed off with a weak solution of carbolic acid. This treatment was continued until the tenth day, when the limb was removed from the fracture box, and suspended on the anterior splint of N. R. Smith. This apparatus was found to answer an admirable purpose in affording steady and equable support to the limb, while it relieved the sloughing surface on the posterior aspect of the leg from the heat and pressure which we had been unable to prevent while the fracture box was used. It also enabled us to constantly observe the progress of the sloughing process, and to apply our dressings without difficulty. The appearance of the leg at this time was anything but encouraging; although the wound leading to the broken fragments had apparently closed up, yet the extensive sloughing of the integument, which included the greater portion lying between the points mentioned as the limit of discoloration, *viz.:* from a point two inches below the seat of fracture to one four inches above the same, rendered the leg, within these limits, an almost entire mass of disease.

The splint used was of wood, as we were unable to procure the wire splint recommended by Dr. Smith. It was carefully adapted to the length of the limb, and to the angles of the knee and foot, with the limb slightly flexed at the knee-joint, and extending about six inches above the knee. Thus adapted, it was laid along the anterior surface of the leg, and confined by strips of adhesive plaster: one around the foot, one around the ankle, just below the edge of the sloughing surface, one just above the same, one around the knee, and one above the knee. The hooks attached to the suspensory cord were then applied, and the limb swung up, and a roller bandage applied around the limb and splint, from the toes to the ankle, and another from the upper edge of the slough to the end of the spint above the knee.

Carbolic acid dressings were used throughout the whole course; and were prepared in the manner advised by Professor E. Andrews, in his article on antiseptic surgery, in the Chicago MEDICAL EXAMINER, for December, 1869. The carbolic oil and the five per cent. solution were used in the following manner: the sores, after being thoroughly cleansed, were syringed off with the five per cent. solution; a piece of patent lint wide enough to embrace the whole of the sloughing surface was then saturated with the carbolic oil, and wrapped around the limb, and held in this situation by a many-tailed bandage, passed under the leg and tied over the splint above. These dressings proved in every way satisfactory; and throughout the whole period in which the patient was under our care, the improvement so far as the leg was concerned, was almost uninterrupted.

On the eighteenth day, *viz.:* Dec. 25th, symptoms of tetanus appeared; the patient complaining of stiffness and soreness of the jaw and neck, and inability to open the mouth more than half way. Bromide of potassium and extract of Indian hemp were prescribed, in the proportion of twenty grs. of the former with two grs. of the latter, every four hours. During the three days following the patient remained in much the same state; on the 28th, the dose of Indian hemp was increased to four grs. I transcribe from my note book the following condensed record of the case from that date:

*Dec. 29th.* Trismus slightly increased; substituted the tr. of Indian hemp for the solid extract, and ordered half a drachm with 40 grs. brom. potass. every five hours.

*30th.* No change.

*31st.* Trismus slightly increased.

*Jan. 4th.* Has remained in about the same condition since the 31st ult.

*5th.* Trismus considerably increased, with some stiffness of the muscles of the back, and jerking of the injured leg. Head drawn back slightly; appetite poor; hands and feet cold; pulse 100. The bromide of potassium and tr. of hemp were now discontinued, and as the bowels were confined, an ounce of castor oil, with half a drachm of turpentine, administered, and one forty-eighth gr. sulphate of atropia injected into the arm. At 5 P.M. there was marked improvement; extremities warm, skin moist, pulse 90, and suffering no pain; pupils not affected. As the bowels had not yet moved, the castor oil was repeated.

*6th.* Trismus about the same; had some "jerking" of the leg during the night; pulse 110; bowels moved this morning. One forty-eighth gr. of atropia was injected.

*7th.* Patient not so well; trismus somewhat increased, with more "jerking" of the leg than heretofore; abdominal muscles rigidly contracted; extremities cold; pulse 110; pupils normal. One thirtieth gr. of atropia was injected, and repeated at 5 P. M., and again at 12 P.M.

*8th.* Not much change. The leg still "jumps;" abdominal muscles rigidly contracted, and there are occasional spasms of pain and cramping of the bowels. Bowels confined; pulse 98; pupils dilated. Administered 10 grs. hydg. chol. mite., to be followed in four hours by an ounce of castor oil, with half a drachm of turpentine, and injected one forty-eighth gr. sulphate of atropia. At 8 P.M. the injection was repeated.

*9th.* Is feeling about the same as yesterday. Bowels moved during the night; pulse 100; pupils dilated. One forty-eighth gr. atropia was injected, and at 8 P.M. one thirtieth gr.

*10th.* Can observe no change. Injected one forty-eighth gr. At 8 P.M. an ounce of castor oil was administered, and the injection of atropia repeated.

*11th.* About the same as yesterday. Bowels moved this morning; pulse 95; pupils only slightly dilated; one thirtieth gr.

sulphate atropia was injected, and at 8 P.M. one twentieth gr.

*12th.* Rested well last night, and feels better this morning. Injected one thirtieth grain of atropia, and at 8 P.M. one twentieth gr.

Our patient continued in about the same state until the 19th, the abdominal muscles remaining perfectly rigid, and the trismus marked, though not interfering greatly with deglutition, nor giving rise to much pain. The appetite fair and the pulse ranging from 90 to 110. Laxatives were administered occasionally, as required, while the amount of atropia injected was gradually increased, until one twelfth gr. was injected twice daily.

*19th.* Patient appears a good deal worse than usual. Trismus somewhat increased; abdomen perfectly hard, with a great deal of pain and cramping. There is more "jerking" of the affected limb than heretofore; and the respiratory muscles seem to be implicated. He has some difficulty of breathing, and is unable to fully inflate the lungs. Bowels open; appetite poor; pulse 110; pupils only slightly dilated. One twelfth gr. atropia injected, and repeated at 8 P.M.

*20th.* About the same as yesterday. Injections repeated.

*21st.* Is a good deal better. Rested well through the night. Trismus somewhat decreased; abdominal muscles much less rigid, and there is no jerking of the leg. Pulse 90; pupils partially dilated. One twelfth grain of atropia was injected, and repeated at 8 P.M.

*22d.* The improvement noticed yesterday still continues. Trismus much less marked, and the rigidity of the abdomen almost disappeared. There is no pain nor cramping, and the respiration is easy and natural. Appetite good; bowels open; pulse 90; pupils dilated. One twelfth gr. atropia was injected, and at 8 P.M. one twenty-fourth gr.

From this time forward the improvement was uninterrupted and rapid, the tetanic symptoms disappearing with so much celerity that on the 24th there remained no apparent trace of the disease. The injections of atropia were discontinued on the 26th, and on the same day the splint was removed, and the leg found to be of proper length and shape, and the bones firmly united. At this time, Feb. 4th, the external sores are less than one-third their original size, and fast filling up. During the whole course of the treatment the patient was allowed the most generous diet that could be procured.

